# Photoallergy to Naproxen

**DOI:** 10.7759/cureus.18961

**Published:** 2021-10-22

**Authors:** Patricia Rojas Perez-Ezquerra, Ines Torrado-Español, Melina Tejero-Alcalde, Cristina Cuevas-Bravo, Blanca Noguerado-Mellado

**Affiliations:** 1 Allergy, Hospital General Universitario Gregorio Marañon, Madrid, ESP

**Keywords:** naproxen, photoallergy, photodematosis, patch tests, photo patch tests

## Abstract

Reactions caused by photosensitivity, also called photodermatosis, are cutaneous reactions induced or exacerbated by exposure to electromagnetic radiation, including UV radiation, visible light, and infrared radiation. We present the case of a 41-year-old man with no personal history of allergy and who is referred to our Drug Allergy Unit for study. We performed a conventional patch test (non-irradiated) and photopatch (with the application of UVA) with reading at 48 and 96 hours and 24 hours after irradiation with an intensity of 5J/cm2. Drug-induced photosensitivity can manifest itself in two clinically indistinguishable forms: photoallergy and phototoxicity. Photoallergic reactions are due to an immunological response of type IV hypersensitivity (a cell-mediated mechanism). We present a case of photoallergy due to sensitization to naproxen, confirmed by photopatch tests.

## Introduction

Reactions caused by photosensitivity, also called photodermatosis, are cutaneous reactions induced or exacerbated by exposure to electromagnetic radiation, including ultraviolet radiation (UVR), visible light, and infrared radiation. They have been classified into four entities with well-defined characteristics: immunologically mediated photodermatosis or idiopathic etiology, photosensitivity induced by exogenous or endogenous drugs/chemical agents, hereditary photodermatosis due to alteration in DNA repair or chromosomal instability, and photographed dermatosis [1]. The photosensitivity reactions can be further divided into phototoxic and photoallergic.

Clinically, photoallergic reactions are pruritic, eczematous eruptions in sun-exposed areas of skin that develop 24 to 48 hours after sun exposure. Similarities in symptomatology may make the diagnosis difficult.

Many drugs are involved in photosensitivity reactions; the majority of drug-induced photosensitivity reactions are phototoxic. Common drugs include tetracyclines, hydrochlorothiazide, sulfonamides, fluoroquinolones, phenothiazines, and in the case of nonsteroidal anti-inflammatory drugs (NSAIDs), the most frequent drugs involved are piroxicam and ketoprofen [2]. Naproxen is an NSAID belonging to the family of arylpropionic acids; few cases of photoallergy have been reported with this drug.

## Case presentation

**Figure 1:**
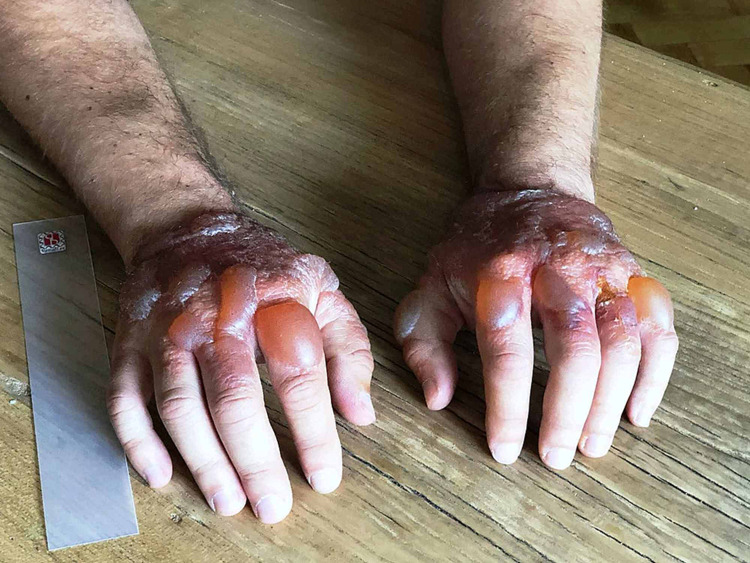
Slight bullous lesions on the back of both hands

We present the case of a 41-year-old man with no personal history of allergy and who is referred to our Drug Allergy Unit for study. The patient had presented 10 hours after taking 500 mg of naproxen and being exposed to the sun in a mountain area, bullous slightly lesions, painful without pruritus, on the back of both hands, respecting the rest of the tegument, which he had covered (Figure 1). He had not taken other medications and had not applied any external agent in the area of ​​appearance of the lesions. He had previously tolerated naproxen and other arylpropionic acids or other NSAIDs. Treatment with topical corticosteroid was started with complete resolution of the skin lesions in three months, without subsequent desquamation or residual hyperpigmentation.

Given the suspicion of photosensitivity reaction by naproxen, we performed a conventional patch test (non-irradiated) and photopatch (with the application of UVA) with reading at 48 and 96 hours and 24 hours after irradiation with an intensity of 5J/cm2.

**Figure 2:**
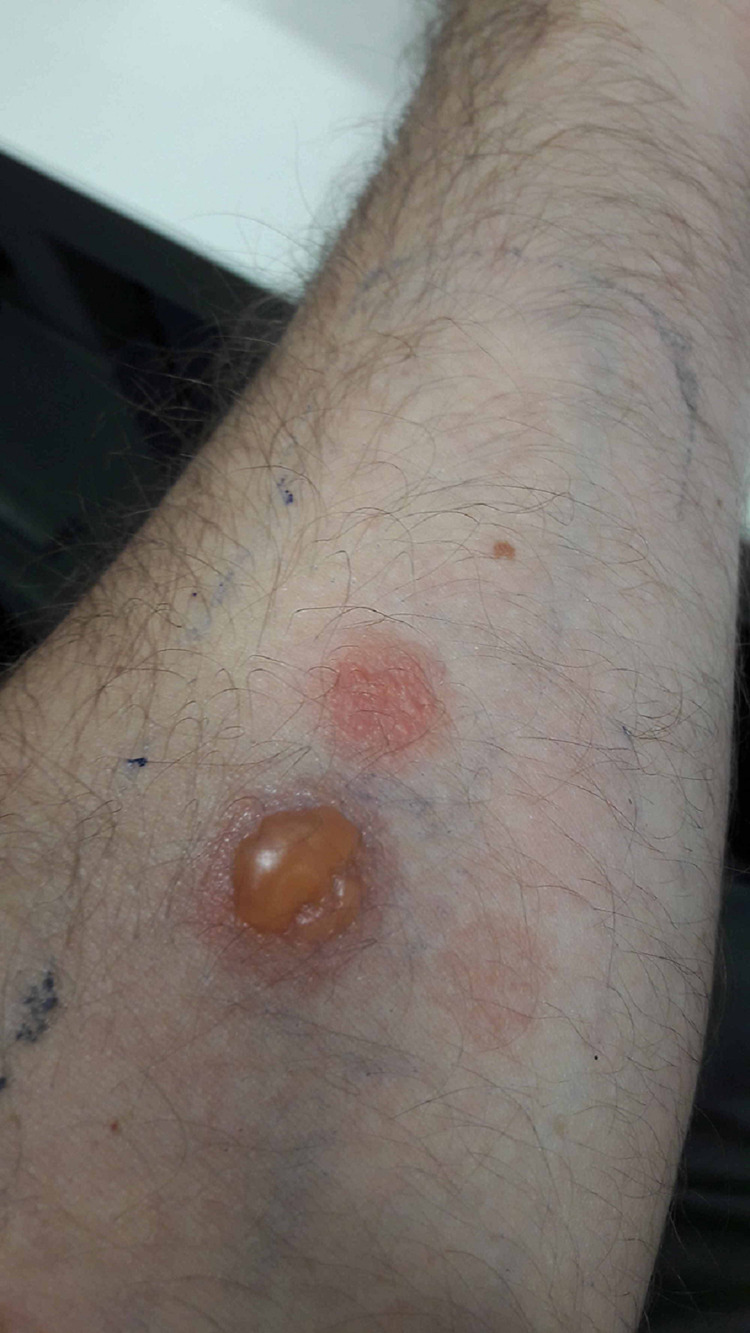
Positive photopatch test at 48 hours with naproxen (+++) and piketoprofen (++)

The tests were performed on both forearms with two identical series of allergens, according to the recommendations of the clinical guidelines [3]. We tested: naproxen 5% in petrolatum (pet), ibuprofen 5% pet, Dexketoprofen 3.6% pet, and 2.5% pet ketoprofen, as well as 10% nabumetone in dimethylsulfoxide (DMSO)-NSAID, which although not belonging to the arylpropionic family has a chemical structure very similar to naproxen. In addition, control was made with DMSO, a vehicle that can sometimes be irritating. The photopatch tests were positive at 48 h with naproxen (+++), piketoprofen (++), and nabumetone (+) at 48 hours and 96 hours. Negative with the rest (Figure 2). The conventional patch tests were negative at 48 and 96 hours with all the drugs.

## Discussion

Drug-induced photosensitivity can manifest itself in two clinically indistinguishable forms: photoallergy and phototoxicity.

Phototoxicity is the most frequent form. It is a non-immunological reaction that is produced by the direct interaction of a drug (chromophore) and the appropriate radiation [4]. It can occur in any exposed individual, at the appropriate doses of the agent, and through radiation that activates it. It does not need a period of sensitization; it can appear after the first exposure. The photosensitizing agent, once activated by UVR, directly damages the skin. The onset period is within minutes to hours after exposure to the agent + UVR.

On the other hand, the photoallergic reactions are due to an immunological response, so they can only occur after an individual has already been sensitized to the agent, and they typically develop 24-72 hours after the exposure. These occur through a cell-mediated immune response (type IV hypersensitivity reaction) that is independent of the dose of drug or amount of UVR received. When the photosensitizer absorbs photons from UVR, the energized molecule can bind proteins in the skin and form new antigens [5]. These antigens are then processed by Langerhans cells and presented on the major histocompatibility complex (MHC) II to activate T-cells, which migrate to the skin to execute an immune response.

A complete allergy workout must be performed to know the type of the reaction, as important consequences can occur in the future to the patient, which is another exposition to the culprit drug.

## Conclusions

In summary, we present a case of photoallergy due to sensitization to naproxen, confirmed by photopatch tests. All anti-inflammatories of the arylpropionic group were banned to avoid reactions in the future.
